# The Cuban scorpion *Rhopalurus junceus* (Scorpiones, Buthidae): component variations in venom samples collected in different geographical areas

**DOI:** 10.1186/1678-9199-19-13

**Published:** 2013-05-20

**Authors:** Rodolfo Rodríguez-Ravelo, Fredy I V Coronas, Fernando Z Zamudio, Lidia González-Morales, Georgina Espinosa López, Ariel Ruiz Urquiola, Lourival D Possani

**Affiliations:** 1 Center for Mountain Development, Ministry of Science, Technology and Environment, Limonar de Monte Roux, El Salvador Guantánamo, Cuba; 2 Department of Molecular Medicine and Bioprocesses, Biotechnology Institute, National Autonomous University of Mexico, Cuernavaca, Mexico; 3 Department of Biochemistry, School of Biology, University of Havana, Havana, Cuba; 4 Center of Marine Research, University of Havana, Havana, Cuba

**Keywords:** Cuba, Hyaluronidase, Peptides, Phospholipase, Proteomics, *Rhopalurus junceus*, Scorpion

## Abstract

**Backgound:**

The venom of the Cuban scorpion *Rhopalurus junceus* is poorly study from the point of view of their components at molecular level and the functions associated. The purpose of this article was to conduct a proteomic analysis of venom components from scorpions collected in different geographical areas of the country.

**Results:**

Venom from the blue scorpion, as it is called, was collected separately from specimens of five distinct Cuban towns (Moa, La Poa, Limonar, El Chote and Farallones) of the Nipe-Sagua-Baracoa mountain massif and fractionated by high performance liquid chromatography (HPLC); the molecular masses of each fraction were ascertained by mass spectrometry analysis. At least 153 different molecular mass components were identified among the five samples analyzed. Molecular masses varied from 466 to 19755 Da. Scorpion HPLC profiles differed among these different geographical locations and the predominant molecular masses of their components. The most evident differences are in the relative concentration of the venom components. The most abundant components presented molecular weights around 4 kDa, known to be K^+^-channel specific peptides, and 7 kDa, known to be Na^+^-channel specific peptides, but with small molecular weight differences. Approximately 30 peptides found in venom samples from the different geographical areas are identical, supporting the idea that they all probably belong to the same species, with some interpopulational variations. Differences were also found in the presence of phospholipase, found in venoms from the Poa area (molecular weights on the order of 14 to 19 kDa). The only ubiquitous enzyme identified in the venoms from all five localities studied (hyaluronidase) presented the same 45 kD molecular mass, identified by gel electrophoresis analysis.

**Conclusions:**

The venom of these scorpions from different geographical areas seem to be similar, and are rich in peptides that have of the same molecular masses of the peptides purified from other scorpions that affect ion-channel functions.

## Background

Scorpion venoms are very diverse in chemical composition and action modes, but are known to contain enzymes, peptides, carbohydrates, lipids, biogenic amines and many other components [1-4]. Venom lethality among scorpions of the family Buthidae is attributed to the presence of toxic peptides that recognize ion channels, mainly Na^+^ and K^+^[5-7]. The most important peptides for toxicity are the alpha- and beta-scorpion toxins (here abbreviated α- and β-NaScTx), which are specific for Na^+^ channels. Two recent reviews on the subject can be found in Gurevitz [8] and Pedraza-Escalona and Possani [9].

Studies on the bioactive components of Cuban scorpions are scarce. As to the venom of *Rhopalurus junceus* (here thereafter abbreviated *R. junceus*), only a general biochemical and molecular biological characterization has been reported with some details [10]. The fact that this species is known to occur in different geographical locations in Cuba has instigated the present study.

Since venom contains a mixture of peptides and proteins secreted by specific glands, an analysis of venom components can produce a valuable fingerprint that is useful as a reference tool in taxonomic analysis, as a complementary method for morphological and behavioral characterization, but also for species identification and classification of related specimens [11].

Intraspecific venom variation has been previously studied in scorpions [12-15], and extensively in snakes [16,17]. As well as being of interest in the study of the ecology and evolution of venomous animals, this phenomenon is relevant to therapy for scorpion stings and snakebites. Scorpion and snake venoms also represent a valuable natural source of biological products for basic research and biomedicine [16]. Thus it is useful to determine whether the components of interest are more abundant in the venom of certain individuals than those of others.

Venom composition variability among some scorpion species has been examined for such purposes as ascertaining measurable differences in the concentration of α-NaScTx between individual *Tityus serrulatus* scorpions of Brazil [18].

More recently, venom variability in specimens of the scorpion *T. serrulatus* was assessed by matrix-assisted laser desorption ionization time-of-flight mass spectrometry (MALDI-TOFMS) analyses, by which significant variations were observed in the venom from a single individual extracted at different times, especially in later extraction events [13]. These variations are most likely related to production dynamics in venom gland cells. Equivalent results have been found in the venom of the scorpion *Androctonus mauretanicus*, whose lethality varies from specimen to specimen and pharmacokinetic parameters differ markedly in venom and antivenom [19].

Badhe *et al*. [20] detected intraspecific protein diversity using sodium dodecyl sulfate polyacrylamide gel electrophoresis (SDS-PAGE) in venom from the red scorpion *Mesobuthus tamulus* from western and southern India. The authors suggested that differences in the band patterns of separated proteins in all venom samples indicated the existence of genetic variation in venom production among scorpion strains of western and southern India. Omran and McVean [12] examined intraspecific variations in venom from the scorpion *Leiurus quinquestriatus* collected in Egypt (Sinai and Aswan Deserts). SDS-PAGE and a densitometric gel scan showed that venom originating from Aswan contains several protein bands that are absent from Sinai-sourced venom. In contrast Sinai venom appears to have a large proportion of protein in the molecular weight range known to include toxins.

Proteomic analyses show that single scorpion venom might contain more than 100 peptide components [21,22]. Out of more than 1,500 different species of scorpions known to exist in the world, approximately 50 have been characterized either by proteins or individual peptides [5,6,23]. Approximations approach nearly 100,000 for distinct components in scorpion venom peptides [7]. An estimative from 2006 shows that no more than 350 components were known at that time [7]. However, updated information available in the Animal Toxin Annotation Program of UniProt (http://www.uniprot.org/program/Toxins) provided manually curated protein entries for approximately 750 scorpion venom proteins.

A number of hyaluronidases have been isolated from the venoms of bees, snakes, wasps, hornets, spiders and fishes [24-31]. Furthermore, hyaluronidases have also been purified from the venoms of a few scorpion species: *Heterometrus fulvipes*, *Tityus serrulatus*, *Palamneus gravimanus* and *Buthus martensi*[32-35]. The only hyaluronidase sequence information obtained from scorpion (*Tityus stigmurus*) venom indicates a lack of similarity to any other known protein sequences [36]. However, studies so far have focused on the low-molecular-weight toxins, probably due to their apparent medical/biological significance, whereas the high-molecular-weight toxins or proteins have been scarcely studied thus far.

The comparison of sufficiently representative venom variations among scorpions from different geographical areas might be useful for taxonomic differentiation, as was found between the American scorpions of the species *Centruroides sculpturatus* and *Centruroides exilicauda*, initially thought to be synonymous species [37]. The study conducted by the latter authors should be taken into consideration to ascertain whether the differences between individuals identified as a single species but collected in different geographical areas might support a different taxonomic assignation.

In relation to the venom of *R. junceus*, the scorpion of interest in the present communication, there are several reports dealing with its toxicity and pharmacology, electrophoretic and chromatographic separation, antimicrobial activity and general biochemical characterization [10,38-43].

This scorpion species is equally distributed throughout Cuba, including the islands adjacent to the country. It is the most common scorpion in the country, and stings hundreds of people each year, but its venom contains an LD50 dose of about 8.0 mg/kg, which is not dangerous to humans (http://en.wikipedia.org/wiki/Rhopalurus_junceus). No deaths have been reported, with the exception of a few cases most likely due to anaphylactic shock following a hypersensitivity immune reaction [38].

Apart from its bioactive peptides, the venom also displays activities of such enzymes as hyaluronidase and phospholipase. While the hyaluronidase activity appears ubiquitous in all venom samples collected in distinct geographical areas showed this activity, phospholipase activity was observed in scorpions collected in the humid area of Baracoa, Guantanamo Province.

In the present communication, the venoms of scorpions collected in five different locations were studied after chromatographic separation and proteomic analysis. Despite the important morphological similarities found, the different biochemical and mass spectrometry results indicate that we might be dealing with very important interpopulational variations, or even with different subspecies of the *R. junceus* scorpion found in the five populations of the Nipe-Sagua-Baracoa mountain massif. Among a total of 227 molecular masses identified from the five different geographical areas studied, we identified 153 as different components.

## Methods

### Sources of venoms and chemicals

Scorpions of the species *R. junceus* were collected in five different locations, with official permission (CITMA 007.01.126) of the “Centro de Inspección y Control Ambiental” of Cuba for the collection and management of flora and fauna.

The *R. junceus* venom was obtained by electrical stimulation from ten adult scorpions from five different geographical areas of Cuba. From Moa: nine females and one male, from La Poa: six females and four males, in Limonar: seven females and three males, from El chote: eight females, two males, and in Farallones: seven females, three males.

After three days in captivity, the individual scorpions from each area were milked and pooled together, with the venom secretion having been collected inside small ampoules and lyophilized. The venom was kept at −20°C. Once in Mexico, the content of each tube was solubilized in water, centrifuged at 10,000 × *g* for 15 minutes; and the absorbance of the soluble venom was read at 280 nm. The soluble venom (assuming one unit of absorbance is equivalent to 1 mg/mL of venom concentration) was adjusted to a final concentration of about 2 mg protein in 300 μL, and applied to the HPLC column for each independent chromatographic separation. The solvents and chemicals used were of analytical grade and double-distilled water was used throughout [10,21].

### Isolation procedures

The soluble venom from each locality was separately applied into the high performance liquid chromatographic (HPLC) system. Usually, 2 mg dissolved in about 100 μL was applied each time. An analytical C18 reverse-phase column (dimensions of 250 × 10 mm) obtained from Vydac (USA) was used for HPLC fractionation. Components were purified using a linear gradient from 0% solution A [0.12% trifluoroacetic acid (TFA) in water] to 60% solution B (0.10% TFA in acetonitrile), run for 90 minutes. The detection was monitored by absorbance at 230 nm with 0.5-unit sensitivity and eluted at 1 mL/min flow-rate. Fractions were collected manually and dried using a Savant Speed-Vac drier.

### Mass spectrometry analysis

For fingerprint analysis, the approximate amount of protein content of each dried sample was estimated, based on the HPLC profile of the separation (integral under the curve for each component). The samples were reconstituted to a final concentration of 0.1-0.5 μg/μL of 50% acetonitrile with 1% acetic acid and directly applied into a Finnigan LCQ^Duo^ ion trap mass spectrometer (USA), using a Surveyor MS syringe pump delivery system. The eluate at 10 μL/min was split to allow only 5% of the sample to enter the nanospray source (0.5 μL/min). LC-MS/MS analyses were performed with a PicoFrit needle/column RP C18 from New Objective (USA) using a fast gradient system from 0 to 60% of B (acetonitrile and acetic acid 1%) in 60% minutes. The spray voltage was set between 1.6 and 1.8 kV while the capillary temperature was set from 100 to 130°C depending on the chosen experimental conditions. All spectra were obtained in the positive-ion mode. The data acquisition and the deconvolution of data were performed with the Xcalibur software on a Windows NT PC system.

### Hyaluronidase activity

The activity of the enzyme hyaluronidase was determined using two different methods described, respectively, by Tolksdorf *et al*. [44] and by Cevallos *et al*. [45]. The first protocol is known as the turbidimetric method, in which hyaluronic acid (0.4 mg/mL) is dissolved in 100 mM Na-phosphate buffer, pH 5.8, containing 150 mM NaCl and hexadecilpiridinium made at 10% concentration in distilled water. Small amounts (2–4 μg) of either venom or semi-purified fractions were used in each tube. The turbidity was read at 540 nm. The method by by Cevallos *et al*. [45] employed sodium dodecyl sulfate polyacrylamide gel electrophoresis (SDS-PAGE) [46], where the acrylamide was copolymerized with 0.5 mg/mL hyaluronic acid. The apparent molecular weight of the enzyme, if present, was observed by a clear degraded zone in the position of the SDS-PAGE gel where the enzyme had migrated, after staining and washing.

### Phospholipase activity

Phospholipase activity of soluble venoms was determined by incubating aliquots of the samples into the gel-plate medium containing the appropriate substrate, as described by Habermann *et al*. [47]. This is a sensitive and very simple assay, consisting of a plate containing egg yolk (rich source of phospholipids) in a solid medium of 0.3% agarose in the presence of 10 mM calcium, and observation of the clear extension around the point at which the samples are applied, after a certain number of hours. For the entire venom, at least five-hour incubation is required. A known phospholipase is always used as positive control and buffer alone as negative control.

## Results and discussion

**Figure 1:**
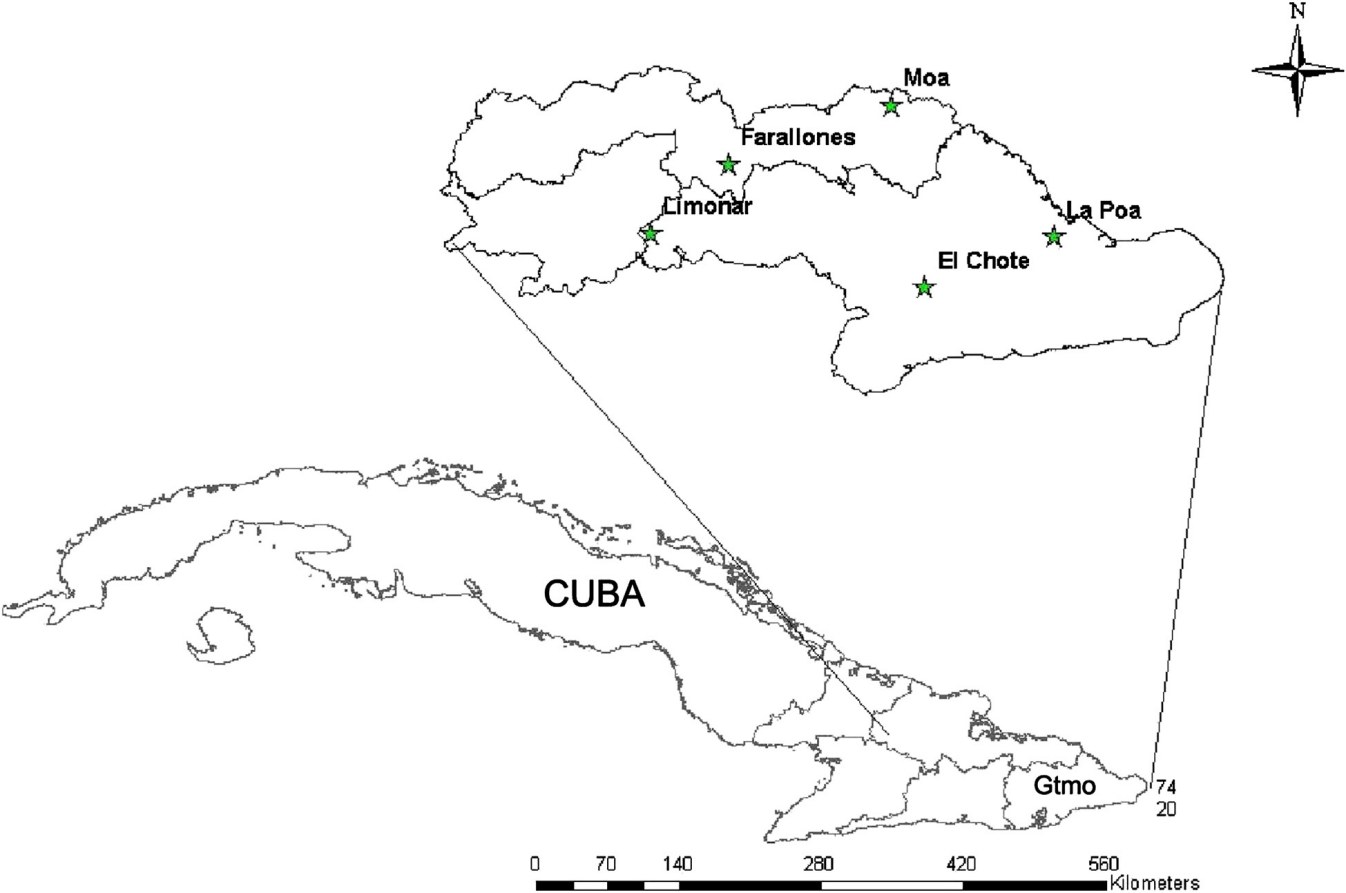
Map of Cuba showing the enlarged area (Guantanamo - Gtmo) were samples were collected.

Figure 1 shows a map corresponding to five geographical areas where scorpions judged to be of the species *R. junceus*, based on various morphological markers, were collected. Ten animals from each locality were milked for venom, as described in the section Material and Methods, after which the samples were subjected to chromatographic separation and molecular mass determination.

**Figure 2:**
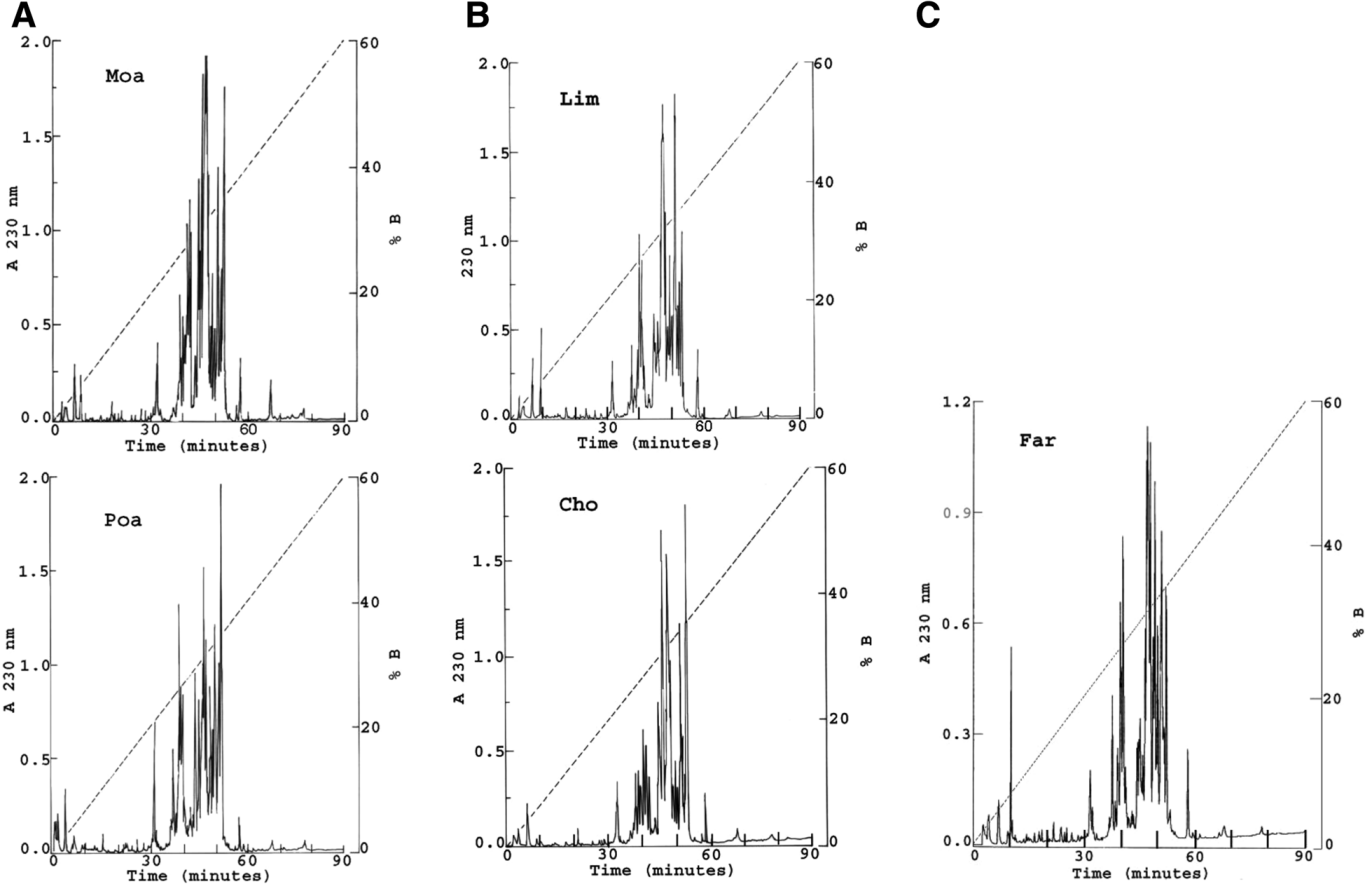
**HPLC separation of soluble venom from different geographical areas:** A 2-mg protein portion from each venom was separated into a C18 reverse-phase column eluted with a linear gradient from solution **A** (0.12% TFA in water) to 60% solution **B** (0.10% TFA in acetonitrile), run for 60 minutes: (**A**) venom of scorpions collected in Moa and La Poa; (**B**) venom collected in Limonar and El Chote; (**C**) venom collected in Farallones.

Figure 2 shows the profile, obtained by chromatographic separation, of the soluble venoms in a C18 reverse-phase column. For better clarity, the various components resolved by this technique were divided into three separate sections of the figure: (A) venoms collected in Moa and La Poa (abbreviated Poa); (B) scorpions from the area Limonar (Lim) and El Chote (Cho), and (C) the venom obtained from animals collected in Farallones (Far). Close analysis of these five separations reveals similar characteristics.

The venoms do contain some closely related profiles in relation to the major components and time of elution, but the relative concentrations seem to be variable. This is already well documented from similar analyses conducted on other venoms, especially when the number of individuals used for preparation of the pooled mixture is small [48,49].

During three decades, the work performed in our laboratory on venoms collected from scorpions of the species *Centruroides noxius* and *Centruroides limpidus limpidus*, collected by the thousands every year (usually four to five thousand annually), has always shown a very similar separation profile using Sephadex G-50 and Ion-exchange columns, also confirmed when using HPLC [50,51]. Some of the differences reported herein might well be due to the number of specimens used, but also to the different number of males and females in a given sample.

It has been reported that some differences are found when scorpion venoms are fractionated separately for each sex of the same species [52,53]. Nevertheless, it is evident that the majority of the venom components are eluted in 30 to 60 minutes, regardless of the sample origin. They do contain components that elute quite similarly in a C18 reverse-phase column. Peptides eluting around 30–40 minutes from other venoms of scorpions of the family Buthidae correspond to molecular masses close to 4 kDa, and have been demonstrated to be peptides that recognize K^+^ channels, whereas peptides eluting in 40 to 50 minutes (the most abundant components of the venom) usually correspond to a molecular weight of approximately 7 kDa and have been shown to be specific for Na^+^ channels [5,6,36].

These results are also in agreement with a previous communication from our laboratory dealing with the biochemical and molecular biological characterization of *R. junceus* venom [10]. In the latter publication the presence of 16 different peptides in the range of 7–9 kDa were cloned and reported, among which, are certainly the Na^+^-channel toxins. In the same publication, physiological experiments showed the presence of both types of peptides in this venom: those affecting Na^+^ and those affecting K^+^ channels, especially hERG potassium channels.

As shown in Figure 2, the number of subfractions separated according to geographic areas is on the order of 60, all of which were submitted to mass spectrometry analysis.

Table 1 displays the results of the proteomic analysis of the fractions collected from the five HPLC columns, using the same operator, column and apparatus. Furthermore, it can be noted that the elution times of the various components are highly uniform, varying less than one minute, as reflected in the clustering in Table 1.

**Table 1:** Retention times and molecular masses of venom components

**RT (min)**	**Moa MW Da**	**La Poa MW Da**	**Limonar MW Da**	**El Chote MW Da**	**Farallones MW Da**
2.84				466	
4.20				2435	
6.89-6.98	5361		3215	6432	5360
8.71-10.24	2173		2173		2174
21.56-21.73				5175	2548
26.26		3554			
30.57-31.35		4036, 3873, 4139, 4195, 3947, 4033	*4033, 4144		
31.51-31.96	3946	4837, 4943	3944		3925
32.07-32.80			*3384, 4940	*3875, 3983, 4143, 3945	*4835, 3963
33.07-34.56	4835		*1922, 4010	*5208, 5769	
36.16-37.03		4199, 4271, 4298, 4048	4271, 4198	6005, 6095	*3360, 4274
37.09-37.54			*4710, 4297, 4209, *4047, 6543	4199, 4297	*4048, 6544
37.92-38.95		7081, 7196	7080	*7192, 7305	7087, *7163, 7053
39.00-39.83	6544, 7080, 7192	7342, 7101	7053, 7085	6242	
40.38-40.65	7037, 7148, 7103	7115, 4184, 4182	*7100, 7265, *7099, 7110	7086	7101
41.16-41.98	7371, 7431		*4181, 7430	*7101, 7375	*4183, 7542, *4871, 4597
42.05-42.83		12735, 7285, 7397, 9264	4467, *6366, 6946	4183,7482	6366
43.22-43.94		3716, 8040	7285,	*6367, 6477, *7394, 7509	
44.17-44.88	7064, 8045, 8200	7039, 7427, 7355	*7031, 7061, 7086, 8042, 7354	7433	7032, 7061, 7090
44.95-45.83	7039	10196, 12236, 14080, 14275, 17140	7355, 7115, *7136, 7079	7034, 7355, 8196	8199, *7136, 7079
46.05-46,74	7518, 7402	7402	7128, 6934	*7040, 7313	7093
46.80-46.99	7431		7179	7058	7128, 7180
47.11-47.33		7973	7431	*7128, 7403	*8081, 7655, 7539
47.51-47.92	7972		*7109, 7971	7431	7973
48.13-49.01	6993, 6891	6860, 13722	6994, 6861	*7109, 7947, 6993	6910, 6861
49.19-49.86	6926	6926, 7162	7960,	6861	*6625, 6994, 7959, 6924
49.93-50.60	7162	14946, 15925, 16923, 18770, 19755	6925	6925	
50.73-51.13	7946		7161		*7160, 6970, *7233, 7039
51.22-51.66		7264, 7280	7041	7162	*14465, 15894
51.79-52.27	7263		7946, 7263	7947	*7263, 7898
52.29-52.91	7280		7280	7280	
53.19-53.52	7949		14560		
54.77-55.19			*6897, 7281	6899	
56.75-58.14	8089	8090	8090	8088	8088

*Each fraction from the HPLC separation shown in Figure 2 was analyzed by mass spectrometry, as described in Material and Methods. When more than one component was found per sub-fraction, the asterisks indicate the component with apparent higher concentration on that particular fraction.

A small time variation can be verified by the identification of identical components found in venoms originating from different areas, which strongly supports the suspicion that the venoms are from the same, or a very closely related, scorpion species. Before analyzing specific examples, it is important to note that the equipment used for mass spectrometry determination (LCQ^Duo^) has effective resolution at the level of one atomic mass unit. Thus, components that differ by about one mass unit can be considered identical.

It is also possible that two peptides with the same molecular masses have different amino acid compositions, and hence are not the same. However, since we are comparing results obtained from scorpions assumed to be of the same species on account of their morphological characteristics, it is very likely that those having the same molecular masses are in fact the same peptides.

Taken together, these observations indicate the presence of about 153 different peptides among the five venoms. It is worth noting that this figure was obtained by adding each of the different masses obtained from the five localities where scorpions were collected. That is, the actual number of distinct peptides found in individual areas is less than 153, out of the 227 total masses obtained.

Consider the following partial list of coincidences which make quite clear that many of the components found have the same molecular masses and come from scorpions of different areas. With an elution time of around 6.9 minutes, a peptide weighing 5360–5361 Da was found in Moa and Far. A peptide of 2173–2174 was found in Moa, Lim and Far with an elution time of around 9 minutes. The same 4033 Da peptide was found in Moa and Lim; another weighing 3944–3945 Da was found in Lim and Cho; one of 4835 Da was present in Moa and Far. Peptides of 4198–4199 Da were found in Moa, Lim and Cho. Peptides eluting in around 37 minutes with molecular masses of 4297–4298 Da were found in Poa, Lim and Cho. Peptides weighing 4047–4048 Da were obtained in Poa, Lim and Far. Peptides of 7080–7081 Da were in Moa, Poa and Lim. Peptides with molecular masses of 7100–7101 were found in Poa, Lim and Cho. Peptides with molecular masses between 4181 and 4283 Da were found in four locations: Poa, Lim, Cho and Far, eluting in about 41 minutes. Peptides of 7354–7355 Da were found in Poa, Lim and Cho. Peptides weighing 7128 were found in Poa, Lim and Far. Peptides of 6993 to 6994 Da were in Moa, Lim, Cho and Far. Peptides with 6924–6926 are in all venoms. Similarly, those having a molecular weight of around 7160–7162 were found in all venoms. Peptides weighing 7263 Da were in Moa, Lim and Cho; others between 7280 and 7281 Da were in Moa, Lim and Far, whereas peptides weighing 8088 to 8089 Da are in Moa, Cho and Far. We have not indicated here all coincidences, but it is quite clear that many of the components found have the same molecular masses and come from scorpions of different areas.

**Figure 3:**
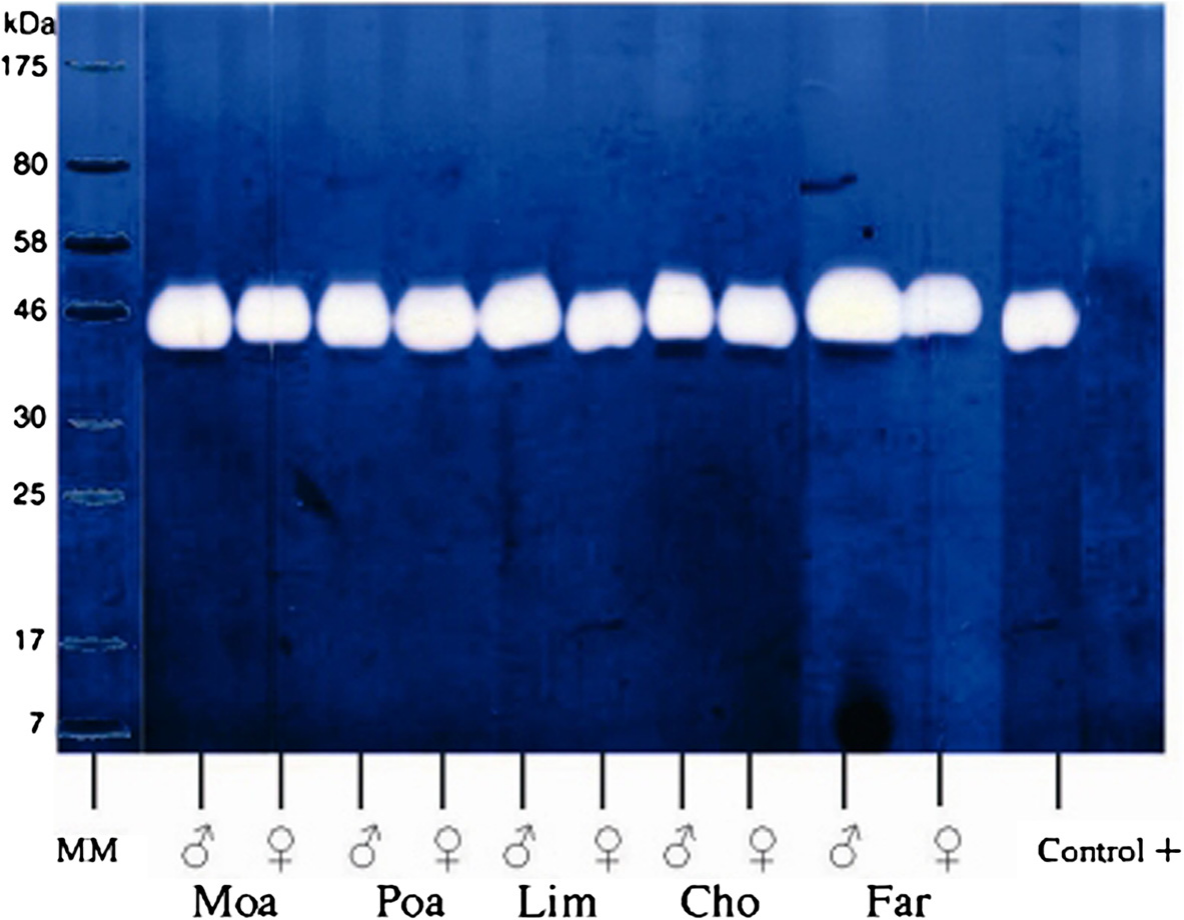
**SDS-PAGE gel electrophoresis for detection of hyaluronidase activity.** Venom from male scorpions (1, 3, 5, 7 and 9) and female scorpions (2, 4, 6, 8 and 19) containing 50 μg of protein each were run in gels containing hyaluronic acid, as described in Materials and Methods. The white band at approximately 46 kDa indicates the presence of hyaluronidase activity. Positive control was a venom sample from the spider *Brachypelma vagans*.

Figure 3 shows the results obtained from separating the five venoms by SDS-PAGE electrophoresis in which hyaluronic acid was included in the gel, for the identification of the enzyme hyaluronidase, as described in the section Materials and Methods. This figure clearly shows that venoms from ten different scorpions, five males (see lanes 1, 3, 5, 7 and 9) and five females (lanes 2, 4, 6, 8 and 10), were shown to contain an active enzyme with a molecular weight of approximately 45 kDa. This activity was also confirmed by the turbidimetric method of Tolksdorf *et al*. [44], using different amounts of venom (data not shown).

Phospholipase activity was verified by the egg yolk-agarose technique (data not shown) in fractions eluting at retention time between 45 and 51 minutes only for the venom obtained in Poa (see molecular masses of Poa in Table 1).

Morphological analyses also performed on specimens collected in the five aforementioned areas strongly support the conclusion that they all belong to the species *R. junceus* (manuscript in preparation).

Taken together, all these results suggest that the scorpions in these five different areas of Cuba are either from the same species or subspecies of *R. junceus*, with the variations already indicated (differences in sex ratio used for milking), or intraspecific variations present in venom of scorpions from other regions [12-15].

It is worth mentioning that the five geographical areas are situated at different altitudes while the mountains arrayed from east to west along the island form a physical barrier between the north and south that gives rise to a climate gradient, leading to more rain in the north in contrast to the semi-desert conditions in the south. There are also two rivers (Sagua in the north) and (Yumurí in the east). Limonar and Farallones are separated by a mountain (460 m) and a river (the Sagua). Farallones and Moa are closely situated, about 20 km apart, at respective altitudes of around 70 and 12 meters above sea level. Furthermore, whereas Farallones has rocky soil, La Moa presents a sedimentary landscape. Limonar is separated from Farallones and Moa by its higher altitude (460 m) and the fluvial river Sagua. El Chote and La Poa are more than 180 Km apart, and are also separated by the rivers Sabanalamar and Yumuri. In addition, El Chote is situated at a much higher altitude (630 m) than La Poa, which is at the sea level. Despite the contribution of these geographical barriers to the variations found in the scorpion venom components from these different areas, the samples are very likely from the same species.

However, the fact that Table 1 shows molecular-weight variations in the components from the different areas leaves the speciation question unresolved. This aspect must be confirmed by means of more appropriate genetic markers. Since the most abundant components are in the range expected for peptides that recognize K^+^ and Na^+^ channels, further efforts should be focused on sequencing and conducting specific physiological assays with these components.

## Conclusion

This communication reports the results of the first proteomic analysis of components from the soluble venom of the Cuban scorpion *Rhopalurus junceus* collected in five different geographical locations of the country. At least 153 different molecular masses compounds were identified, among which are peptides with molecular weights in the order of 4 kDa, known to be K^+^-channel specific peptides, and 7 kDa, known to be Na^+^-channel specific peptides, confirming earlier findings by our group showing the presence of such peptides in this venom [10]. Approximately 30 peptides found in the venom samples from the different geographical areas are identical, supporting the idea that they all probably belong to the same species of scorpion, with some interpopulation variations. The enzyme hyaluronidase is ubiquitously present in all samples, whereas phospholipase activity was found only in scorpions collected in POA. The HPLC separation of the various samples showed similar profile but with some variations, especially in the relative amount of each component.

### Ethical approval

The present study was approved by the Centro de Inspección y Control Ambiental of Cuba for the collection and management of flora and fauna (CITMA 007.01.126).

## Competing interests

The authors declare that there are no competing interests.

## Authors’ contributions

RRR, student from the joint program UNAM-University of Habana, collected the scorpions and made most experiments assisted by specific technicians; FIVC, technician at UNAM, run all the HPLC separations; FZZ, run the mass spectrometry analysis; LGM helped conducted the hyaluronidase assays determination; GEL, tutor from the Habana University program, contributed on the design of experiments, assisted the student and helped writing the article; ARU contributed to the design of experiments, the collection and classification of scorpions; LDP, tutor from the UNAM university, designed the strategy of the work, supervised the conduction of all experiments and corrected the writing of the entire manuscript. All authors read and approved the final manuscript.

## References

[B1] Tu AT Tu AT Scorpion Venoms Venoms: Chemistry and Molecular Biology 1977 New York: John Wiley and Sons 459 483

[B2] Zlotkin E Miranda F Rochat H Bettini S Chemistry and pharmacology of Buthidae scorpion venoms, in Arthrop Venoms, Handbook of Experimental Pharmacology 1978 Berlin: Springer-Verlag 317 369

[B3] Rochat H Bernard P Couraud F Ceccarelli B, Clementi F Scorpion toxins: Chemistry and mode of action Advances in Cytopharmacology 1979 3 New York: Raven Press 325 334 382791

[B4] Possani LD Becerril B Delepierre M Tytgat J Scorpion toxins specific for Na^+^-channels Eur J Biochem 1999 264 2 287 300 10.1046/j.1432-1327.1999.00625.x 10491073

[B5] de la Vega RC R Possani LD Minireview: current views on scorpion toxins specific for K^+^-channels Toxicon 2004 43 8 865 875 10.1016/j.toxicon.2004.03.022 15208019

[B6] de la Vega RC R Possani LD Overview of scorpion toxins specific for Na^+^ channels and related peptides: biodiversity, structure-function relationships and evolution Toxicon 2005 46 8 831 844 10.1016/j.toxicon.2005.09.006 16274721

[B7] Possani LD de la Vega RC R Kastin AJ Scorpion Venom Peptides The Handbook of Biologically Active Peptides 2006 San Diego: Academic Press 339 354

[B8] Gurevitz M Mapping of scorpion toxin receptor sites at voltage-gated sodium channels Toxicon 2012 60 4 502 511 10.1016/j.toxicon.2012.03.022 22694883

[B9] Pedraza-Escalona M Possani LD Scorpion β-toxins and voltage-gated sodium channels: interactions and toxicity effects Front Biosci 2013 18 2 572 587 10.2741/4121 23276943

[B10] García-Gómez BI Coronas FI Restano-Cassulini R Rodríguez RR Possani LD Biochemical and molecular characterization of the venom from the Cuban scorpion *Rhopalurus junceus* Toxicon 2011 58 1 19 27 10.1016/j.toxicon.2011.04.011 21605585

[B11] Newton KA Clench MR Deshmukh R Jeyaseelan K Strong PN Mass fingerprinting of toxic fractions from the venom of the Indian red scorpion, Mesobuthus tamulus: biotope-specific variation in the expression of venom peptides Rapid Commun Mass Spectrom 2007 21 21 3467 3476 10.1002/rcm.3240 17918210

[B12] Omran MA McVean A Intraspecific variation in scorpion Leiurus quinquestriatus venom collected from Egypt (Sinai and Aswan deserts) Toxin Rev 2000 19 3–4 247 264

[B13] Pimenta AM De Marco AF de Lima ME Martin-Eauclaire MF Bougis PE Individual variability in *Tityus serrulatus* (Scorpiones, Buthidae) venom elicited by matrix-assisted laser desorption/ionization time-of-flight mass spectrometry Rapid Commun Mass Spectrom 2003 17 4 413 418 12590389 10.1002/rcm.934

[B14] Borges A García C Lugo E Alfonso M Jowers M Op den Camp H Diversity of long-chain in *Tityus zulianus* and *Tityus discrepans* (Scorpiones, Buthidae): molecular, immunological, and mass spectral analyses Comp Biochem Physiol C Toxicol Pharmacol 2006 142 3–4 240 252 16356783 10.1016/j.cbpc.2005.10.011

[B15] Abdel-Rahman MA Intraspecific diversity of scorpion’s venom and its implication on the pathophysiological effects J Venom Anim Toxins incl Trop Dis 2008 14 1 191 192 10.1590/S1678-91992008000100020

[B16] Chippaux JP Williams V White J Snake venom variability: methods of study, results and interpretation Toxicon 1991 29 11 1279 1303 10.1016/0041-0101(91)90116-9 1814005

[B17] Menezes MC Furtado MF Travaglia-Cardoso SR Camargo AC Serrano SM Sex-based individual variation of snake venom proteome among eighteen *Bothrops jararaca* siblings Toxicon 2006 47 3 304 312 10.1016/j.toxicon.2005.11.007 16373076

[B18] Kalapothakis E Chavéz-Olórtegui C Venom variability among several *Tityus serrulatus* specimens Toxicon 1997 35 10 1523 1529 10.1016/S0041-0101(97)00017-2 9428099

[B19] El Hafny B Chgoury F Adil N Cohen N Hassar M Intraspecific variability and pharmacokinetics, characteristics of *Androctonus mauretanicus* scorpion venom Toxicon 2002 40 11 1609 1616 10.1016/S0041-0101(02)00178-2 12419512

[B20] Badhe RV Thimas AB Harer SL Desphande AD Salvi N Waghmare A Intraspecific variation in protein of red scorpion (*Mesobuthus tamulus*, Coconsis, Pocock) venoms from Western and Southern India J Venom Anim Toxins incl Trop Dis 2006 12 4 612 619

[B21] Batista CV del Pozo L Zamudio FZ Contreras S Becerril B Wanke E Possani LD Proteomics of the venom from the Amazonian scorpion *Tityus cambridgei* and the role of prolines on mass spectrometry analysis of toxins J Chromatogr B Analyt Techol Biomed Life Sci 2004 803 1 55 66 10.1016/j.jchromb.2003.09.002 15025998

[B22] Diego-García E Batista CVF García-Gómez BI Lucas S Candido DM Gómez Lagunas F Possani LD The Brazilian scorpion *Tityus costatus* Karsch: genes, peptides and function Toxicon 2005 45 3 273 283 10.1016/j.toxicon.2004.10.014 15683865

[B23] Fet V Sissom WD Lowe GJ Braunwalder ME Catalog of the scorpions of the world (1758–1998) 2000 New York: New York Entomological Society

[B24] Gmachl M Kreil G Bee venom hyaluronidase is homologous to a membrane protein of mammalian sperm Proc Natl Acad Sci USA 1993 90 8 3569 3573 10.1073/pnas.90.8.3569 7682712 PMC46342

[B25] Xu X Wang XS Xi XT Liu J Huang JT Lu ZX Purification and partial characterization of hyaluronidase from five pace snake (Agkistrodon acutus) venom Toxicon 1982 20 6 973 981 10.1016/0041-0101(82)90099-X 7164113

[B26] Kudo K Tu AT Characterization of hyaluronidase isolated from *Agkistrodon contortrix contortrix* (Southern Copperhead) venom Arch Biochem Biophys 2001 386 2 154 162 10.1006/abbi.2000.2204 11368337

[B27] Girish KS Shashidharamurthy R Nagaraju S Gowda TV Kemparaju K Isolation and characterization of hyaluronidase a “spreading factor” from Indian cobra (*Naja naja*) venom Biochimie 2004 86 3 193 202 10.1016/j.biochi.2004.02.004 15134834

[B28] King TP Lu G González M Qian N Soldatova L Yellow jacket venom allergens, hyaluronidase and phospholipase: sequence similarity and antigenic cross-reactivity with their hornet and wasp homologs and possible implications for clinical allergy J Allergy Clin Immunol 1996 98 3 588 600 10.1016/S0091-6749(96)70093-3 8828537

[B29] Lu G Kochoumian L King TP Sequence identity and antigenic cross reactivity of white face hornet venom allergen, also a hyaluronidase, with other proteins J Biol Chem 1995 270 9 4457 4465 10.1074/jbc.270.9.4457 7876212

[B30] Wright RP Elgert KD Campbell BJ Barrett JT Hyaluronidase and esterase activities of the venom of the poisonous brown recluse spider Arch Biochem Biophys 1973 159 1 415 426 10.1016/0003-9861(73)90469-4 4206202

[B31] Poh CH Yuen R Chung M Khoo HE Purification and partial characterization of hyaluronidase from stonefish (Synanceja horrida) venom Comp Biochem Physiol B 1992 101 1–2 159 163 1499262 10.1016/0305-0491(92)90172-n

[B32] Ramanaiah M Parthasarathy PR Venkaiah B Isolation and characterization of hyaluronidase from scorpion (*Heterometrus fulvipes*) venom Biochem Int 1990 20 2 301 310 2317213

[B33] Pessini AC Takao TT Cavalheiro EC Vichnewski W Sampaio SV Giglio JR Arantes EC A hyaluronidase from *Tityus serrulatus* scorpion venom: isolation, characterization and inhibition by flavonoids Toxicon 2001 39 10 1495 1504 10.1016/S0041-0101(01)00122-2 11478957

[B34] Morey SS Kiran KM Gadag JR Purification and properties of hyaluronidase from Palamneus gravimanus (Indian black scorpion) venom Toxicon 2006 47 2 188 195 10.1016/j.toxicon.2005.10.014 16359718

[B35] Feng L Gao R Gopalakrishnakone P Isolation and characterization of a hyaluronidase from the venom of Chinese red scorpion *Buthus martensi* Comp Biochem Physiol C Toxicol Pharmacol 2008 148 3 250 257 10.1016/j.cbpc.2008.06.003 18611448

[B36] Batista CV Román-González S Salas-Castillo SP Zamudio FZ Gómez-Lagunas F Possani LD Proteomic analysis of the venom from the scorpion *Tityus stigmurus*: biochemical and physiological comparison with other *Tityus* species Comp Biochem Physiol C Toxicol Pharmacol 2007 146 1–2 147 157 17270501 10.1016/j.cbpc.2006.12.004

[B37] Valdéz-Cruz NA Dávila S Licea A Corona M Zamudio FZ García-Valdes J Boyer L Possani LD Biochemical, genetic and physiological characterization of venom components from two species of scorpions: *Centruroides exilicauda* Wood and *Centruroides sulpturatus* Ewing Biochimie 2004 86 6 387 396 10.1016/j.biochi.2004.05.005 15358055

[B38] Cao J Rivera F Bello F Algunos aspectos bioecologicos y farmacológicos del veneno crudo procedente de dos especies de escorpiones cubanos IV Simposio de Zoologia 1997 Habana- Cuba: Livro de Resumenes p. 70

[B39] Pérez Capote M Rodríguez C Guevara I Romeaux R Estudio de toxicidad aguda de las toxinas de los escorpiones *Rophalurus junceus* y *Centruroides gracilis* Rev Cub Farm 2004 38 Supl Esp 156 166

[B40] Hernández-Betancourt O Casado-Hernández I Iglesias-Huerta E Ramírez-Labrada A del Risco-Ramos J Rodríguez-Pargas A Evaluación de la toxicidad *in vitro* del veneno del alacrán *Rophalurus junceus* a través de un ensayo celular Rev Cub Invest Biomed 2009 28 1 1 11

[B41] Hernández-Betancourt O Compte AO Quesada-Leiva L Rodríguez-Pargas A Caracterización electroforética y cromatográfica del veneno del alacrán Rhopalurus junceus Arch Med Camaguey 2009 13 6 1 8

[B42] Díaz-García A Morier-Díaz L Rodríguez-Sánchez H Caballero-Lorenzo Y Fraga-Castro JA Comparison of protein composition and *in vitro* toxicity of venom from cuban scorpions *Rhopalurus junceus* and *Centruroides gracilis* Labiofam 2011 1 21 37

[B43] Rodríguez R Guerra O Baro E Silva J Magdelaine R Acción antimicrobiana del veneno del escorpion *Rhopalurus junceus* (Scorpionida: buthidae) Rev Cubana Farm 2004 38 2 134

[B44] Tolksdorf S McCready MH McCullagh D Schwenk E The turbidimetric assay of hyaluronidase J Lab Clin Med 1949 34 1 74 89 18106411

[B45] Cevallos MA Navarro-Duque C Varela-Julia M Alagon AC Molecular mass determination and assay of venom hyaluronidases by sodium dodecyl sulfate poliacrylamide gel electrophoresis Toxicon 1992 30 8 925 930 10.1016/0041-0101(92)90392-I 1523685

[B46] Laemmli K Cleavage of structural proteins during the assembly of the head of bacteriophage T4 Nature 1970 227 5259 680 685 10.1038/227680a0 5432063

[B47] Habermann E Hardt KL A sensitive and specific plate test for the quantitation of phospholipases Anal Biochem 1972 50 1 163 173 10.1016/0003-2697(72)90495-2 4342994

[B48] Possani LD Fletcher PL Fletcher M Rode GS Mochca-Morales J Lucas S Coronas FV Martin BM Structural and functional characteristics of toxins purified from the venom of the Brazilian scorpion *Tityus serrulatus* Lutz and Mello Mem Inst Butantan 1992 54 35 52

[B49] Schwartz EF Camargos TS Zamudio FZ Silva PL Bloch C Caixeta F Schwartz C Possani LD Mass spectrometry analysis, amino acid sequence and biological activity of venom components from the Brazilian scorpion *Opisthacanthus cayaproum* Toxicon 2008 51 8 1499 1508 10.1016/j.toxicon.2008.03.029 18502464

[B50] Possani LD Dent MA Martin BM Maelicke A Svendsen I The amino terminal sequence of several toxins from the venom of the Mexican scorpion *Centruroides noxius* Hoffmann Carlsberg Res Commun 1981 46 4 207 214 10.1007/BF02906498

[B51] Alagón AC Guzmán HS Martin BM Ramírez AN Carbone E Possani LD Isolation and characterization of two toxins from the Mexican scorpion *Centruroides limpidus limpidus* Karsch Comp Biochem Physiol B 1988 89 1 153 162 2451580 10.1016/0305-0491(88)90277-5

[B52] Yamaji N Dai L Sugase K Andriantsiferana M Nakajima T Iwashita T Solution structure of IsTX: A male scorpion toxin from *Opisthacanthus madagascariensis* (Ischnuridae) Eur J Biochem 2004 271 19 3855 3864 10.1111/j.1432-1033.2004.04322.x 15373831

[B53] De Sousa L Borges A Vásquez-Suárez A Op den Camp HJ Chadee-Burgos RI Romero-Bellorín M Espinoza J De Sousa-Insana L Oscar Pino-García O Differences in venom toxicity and antigenicity between females and males *Tityus nororientalis* (Buthidae) scorpions J Venom Res 2010 1 61 70 21544184 PMC3086188

